# Impact of Environmental Parameters on Marathon Running Performance

**DOI:** 10.1371/journal.pone.0037407

**Published:** 2012-05-23

**Authors:** Nour El Helou, Muriel Tafflet, Geoffroy Berthelot, Julien Tolaini, Andy Marc, Marion Guillaume, Christophe Hausswirth, Jean-François Toussaint

**Affiliations:** 1 IRMES (bioMedical Research Institute of Sports Epidemiology), INSEP, Paris, France; 2 Université Paris Descartes, Sorbonne Paris Cité, Paris, France; 3 Faculté de Pharmacie, Département de Nutrition, Université Saint Joseph, Beirut, Lebanon; 4 INSERM, U970, Paris Cardiovascular Research Center – PARCC, Paris, France; 5 Research Department, INSEP, Paris, France; 6 Hôtel-Dieu Hospital, CIMS, AP-HP, Paris, France; Universidad Europea de Madrid, Spain

## Abstract

**Purpose:**

The objectives of this study were to describe the distribution of all runners' performances in the largest marathons worldwide and to determine which environmental parameters have the maximal impact.

**Methods:**

We analysed the results of six European (Paris, London, Berlin) and American (Boston, Chicago, New York) marathon races from 2001 to 2010 through 1,791,972 participants' performances (all finishers per year and race). Four environmental factors were gathered for each of the 60 races: temperature (°C), humidity (%), dew point (°C), and the atmospheric pressure at sea level (hPA); as well as the concentrations of four atmospheric pollutants: NO_2_ – SO_2_ – O_3_ and PM_10_ (μg.m^−3^).

**Results:**

All performances per year and race are normally distributed with distribution parameters (mean and standard deviation) that differ according to environmental factors. Air temperature and performance are significantly correlated through a quadratic model. The optimal temperatures for maximal mean speed of all runners vary depending on the performance level. When temperature increases above these optima, running speed decreases and withdrawal rates increase. Ozone also impacts performance but its effect might be linked to temperature. The other environmental parameters do not have any significant impact.

**Conclusions:**

The large amount of data analyzed and the model developed in this study highlight the major influence of air temperature above all other climatic parameter on human running capacity and adaptation to race conditions.

## Introduction

Like most phenotypic traits, athletic performance is multifactorial and influenced by genetic and environmental factors: exogenous factors contribute to the expression of the predisposing characteristics among best athletes [1], [2]. The marathon is one of the most challenging endurance competitions; it is a mass participation race held under variable environmental conditions and temperatures sometimes vary widely from start to finish [3]–[5]. Warm weather during a marathon is detrimental for runners and is commonly referenced as limiting for thermoregulatory control [3], [6]. More medical complaints of hyperthermia (internal temperature ≥39°C) occur in warm weather events, while hypothermia (internal temperature ≤35°C) sometimes occurs during cool weather events [3].

In addition, participating in an outdoor urban event exposes athletes to air pollution which raises concerns for both performance and health [7]. Runners could be at risk during competitions as they are subject to elevated ventilation rate and increased airflow velocity amplifying the dose of inhaled pollutants and carrying them deeper into the lungs [7]–[9]. They switch from nasal to mouth breathing, bypassing nasal filtration mechanisms for large particles. Both might increase the deleterious effects of pollutants on health and athletic performance [8], [10]. Exposure to air pollution during exercise might be expected to impair an athlete's performance in endurance events lasting one hour or more [7], [10].

The relationship between marathon performance decline and warmer air temperature has been well established. Vihma [6] and Ely et al. [11], [12] found a progressive and quantifiable slowing of marathon performance as WBGT (Wet Bulb Globe Temperature) increases, for men and women of wide ranging abilities. Ely et al. [13] as well as Montain et al. [14] also found that cooler weather (5–10°C) was associated with better ability to maintain running velocity through a marathon race compared to warmer conditions especially by fastest runners; weather impacted pacing and the impact was dependent on finishing position. Marr and Ely [9] found significant correlations between the increase of WBGT and PM_10_, and slower marathon performance of both men and women; but they did not find significant correlations with any other pollutant.

Previous studies have mostly analysed the performances of the top 3 males and females finishers as well as the 25^th^-, 100^th^-, and 300^th^- place finishers [11], [13]–[16]. Here we targeted exhaustiveness and analysed the total number of finishers in order to quantify the effect of climate on the full range of runners.

The objectives of this study were 1) to analyse all levels of running performance by describing the distribution of all marathons finishers by race, year and gender; 2) to determine the impact of environmental parameters: on the distribution of all marathon runners' performance in men and women (first and last finishers, quantiles of distribution); and on the percentage of runners withdrawals. We then modelled the relation between running speed and air temperature to determine the optimal environmental conditions for achieving the best running performances, and to help, based on known environmental parameters, to predict the distribution and inform runners on possible outcomes of running at different ambient temperatures. We tested the hypothesis that all runners' performances distributions may be similar in all races, and may be similarly affected by temperature.

## Methods

### Data Collection

Marathon race results were obtained from six marathons included in the « IAAF Gold Labeled Road Races » and « World Marathon Majors »: Berlin, Boston, Chicago, London, New York and Paris. From 2001 to 2010 (available data are limited before 2001) the arrival times in hours: minutes: seconds, of all finishers were gathered for each race. These data are available in the public domain on the official internet website of each city marathon, and on marathon archives websites [17] and complementary data when needed from official sites of each race. Written and informed consent was therefore not required from individual athletes. The total number of collected performances was 1,791,972 for the 60 races (10 years × 6 marathons), including 1,791,071 performances for which the gender was known. We also gathered the total number of starters in order to calculate the number and the percentage of non-finishers (runner withdrawal) per race.

Hourly weather data corresponding to the race day, time span and location of the marathon were obtained from “weather underground website” [5]. Four climatic data were gathered for each of the 60 races: air temperature (°C), air humidity (%), dew-point temperatures (°C), and atmospheric pressure at sea level (hPA). Each of these parameters was averaged for the first 4 hours after the start of each race. Hourly air pollution data for the day, time span and location of each race were also obtained through the concentrations of three atmospheric pollutants: NO_2_ – SO_2_ – O_3_ (μg.m^−3^) from the Environmental Agency in each state (the Illinois Environmental Protection Agency for Chicago maratho'n, the Massachusetts Department of environmental Protection for Boston marathon and the New York State Department of Environmental Conservation for New York marathon), and the Environmental agency websites of the three European cities [18]–[20]. All pollutants values were averaged for the first 4 hours after the start of each race.

Concurrent measurements of air pollution for all ten race years (2001–2010) were only available for 3 pollutants, because air pollution monitoring sites typically measure only a subset of pollutants and may not have been operational in all years. In addition, particulate matters PM_10_ were collected in Paris and Berlin, but there were not enough measurements in the other four cities races days.

### Data Analysis and selection

Men and women performances were analysed separately. For each race and each gender every year, we fitted the Normal and log-Normal distributions to the performances and tested the normality and log normality using the Kolmogorov-Smirnov *D* statistic. We rejected the null hypothesis that the sample is normally or log–normally distributed when p values <0.01.

The following statistics (performance levels) were determined for all runners’ performances distribution of each race, every year and for each gender:

the first percentile of the distribution (P1), representing the elite of each race.
the winner.
the last finisher.
the first quartile of the distribution (Q1), representing the 25^th^ percentile of best performers of the studied race.
the median.
the inter quartile range (IQR), representing the statistical dispersion, being equal to the difference between the third and first quartiles.

A Spearman correlation test was performed between each performance level and climate and air pollution parameters, in order to quantify the impact of weather and pollution on marathon performances. Spearman correlation tests were also performed between each environmental parameter. The year factor was not included because we previously demonstrated that for the past ten years, marathon performances were now progressing at a slower rate [21].

### Temperature and running speed

We modelled the relation between running speed of each performance level for each gender and air temperature, using a second degree polynomial quadratic model, which seems appropriate to depict such physiological relations [22]–[24].

The second degree polynomial equation was applied to determine the optimal temperature at which maximal running speed is achieved for each level of performance for each gender, and then used to calculate the speed decrease associated with temperature increase and decrease above the optimum.

We similarly modelled the relation between air temperature and the percentage of runners' withdrawal.

All analyses were performed using the MATLAB and SAS software.

## Results

**Figure 1:**
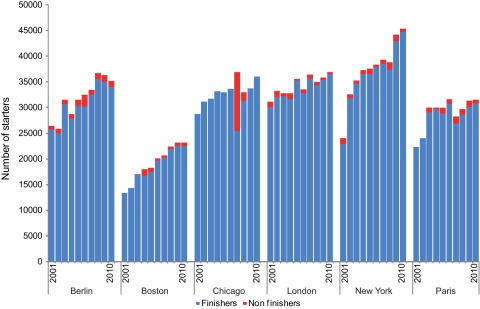
Number of starters and finishers by marathon and year (missing data points for Boston, Chicago and Paris marathons).

The total numbers of starters and finishers of the 6 marathons increased over the 10 studied years (Figure 1). Marathons characteristics are described in supplementary data (Table S1). The race with the least number of finishers was Boston 2001 with 13381 finishers and the highest number was seen in New York 2010 with 44763 finishers.

Three marathons were held in April, the other three during fall. Air temperatures ranged from 1.7°C (Chicago 2009) to 25.2°C (Boston 2004) (Table 1).

**Table 1:** Average and range values of all weather and pollution parameters for the six marathons.

Marathon	Parameter	N	Mean	Std Dev	Minimum	Maximum
**Berlin** Run in September; Starts 9am	Temperature (°C)	10	14.9	3.2	11.3	21.3
	Dew Point (°C)	10	10.6	1.8	5.8	12.3
	Humidity (%)	10	78.0	14.5	55.0	98.5
	Atmospheric pressure (hPA)	10	1017.0	6.3	1003.0	1029.0
	NO_2_ (μg.m^−3^)	10	26.5	4.0	20.8	33.2
	O_3_ (μg.m^−3^)	10	41.0	17.3	21.2	81.8
	PM_10_ (μg.m^−3^)	8	25.1	11.4	7.6	46.5
	SO_2_ (μg.m^−3^)	10	5.0	3.1	1.1	10.7
**Boston** Run in April; Starts 10am	Temperature (°C)	10	11.8	5.1	8.0	25.2
	Dew Point (°C)	10	3.9	3.8	−2.1	10.2
	Humidity (%)	10	62.6	19.9	28.3	91.0
	Atmospheric pressure (hPA)	10	1013.0	12.4	981.6	1029.0
	NO_2_ (μg.m^−3^)	10	29.3	10.3	14.6	50.5
	O_3_ (μg.m^−3^)	10	73.5	25.7	18.5	122.7
	PM_10_ (μg.m^−3^)	0				
	SO_2_ (μg.m^−3^)	10	7.0	2.9	1.6	12.1
**Chicago** Run in October; Starts 7:30am	Temperature (°C)	10	12.1	7.5	1.7	25.0
	Dew Point (°C)	10	4.9	7.6	−5.9	19.0
	Humidity (%)	10	62.8	8.1	52.3	79.2
	Atmospheric pressure (hPA)	10	1022.0	6.4	1012.0	1031.0
	NO_2_ (μg.m^−3^)	10	27.9	13.0	9.7	52.0
	O_3_ (μg.m^−3^)	10	57.1	15.1	35.9	84.0
	PM_10_ (μg.m^−3^)	2	26.7	11.6	15.3	38.0
	SO_2_ (μg.m^−3^)	9	6.5	3.1	2.1	12.4
**London** Run in April; Starts 9:30am	Temperature (°C)	10	12.4	3.2	9.5	19.1
	Dew Point (°C)	10	6.0	2.9	0.8	10.7
	Humidity (%)	10	66.9	16.7	42.9	86.1
	Atmospheric pressure (hPA)	10	1010.0	12.5	976.4	1020.0
	NO_2_ (μg.m^−3^)	10	44.8	14.5	22.8	72.2
	O_3_ (μg.m^−3^)	9	51.4	17.1	35.0	92.3
	PM_10_ (μg.m^−3^)	2	27.8	14.5	13.7	41.9
	SO_2_ (μg.m^−3^)	10	4.5	2.8	0.0	8.8
**New York** Run in November; Starts 10am	Temperature (°C)	10	12.5	4.1	7.1	18.4
	Dew Point (°C)	10	2.3	6.4	−5.6	12.8
	Humidity (%)	10	51.1	12.1	36.5	79.8
	Atmospheric pressure (hPA)	10	1020.0	7.8	1009.0	1034.0
	NO_2_ (μg.m^−3^)	9	55.1	17.2	21.9	77.3
	O_3_ (μg.m^−3^)	10	32.6	12.3	11.1	53.8
	PM_10_ (μg.m^−3^)	10	5.0	0.0	5.0	5.0
	SO_2_ (μg.m^−3^)	9	19.7	12.2	4.8	42.4
**Paris** Run in April; Starts 8:45am	Temperature (°C)	10	9.2	3.2	4.8	17.4
	Dew Point (°C)	10	4.2	4.1	−3.6	13.4
	Humidity (%)	10	72.4	10.1	45.9	85.4
	Atmospheric pressure (hPA)	10	1019.0	6.2	1005.0	1026.0
	NO_2_ (μg.m^−3^)	10	43.0	13.7	23.4	73.1
	O_3_ (μg.m^−3^)	10	66.9	9.8	55.2	82.1
	PM_10_ (μg.m^−3^)	10	37.9	32.6	16.6	132.7
	SO_2_ (μg.m^−3^)	10	6.4	3.7	1.5	12.2

**Figure 2:**
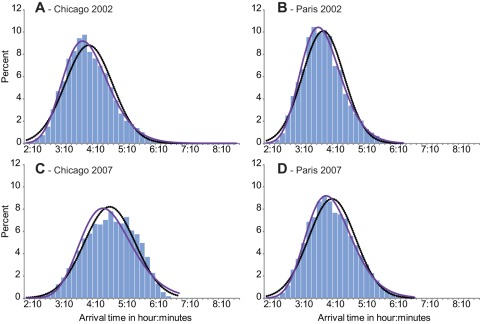
Distribution of performances: example of men's performances distribution for Chicago (in 2002: T°C = 5.4°C; and in 2007: T°C = 25°C); and Paris (in 2002: T°C = 7.6°C; and in 2007: T°C = 17.4°C).

### Performance distribution

For all 60 studied races, the women and men's performance distributions were a good approximation of the “log normal” and “normal” distributions (p-values of Kolmogorov-Smirnov statistics ≥0.01).

Figure 2 illustrates examples of 4 races' performances distribution fit: men's performances distribution of two races in Paris (2002: T° = 7.6°C; and 2007: T° = 17.4°C) and Chicago (2002: T° = 5.4°C; and 2007: T° = 25°C).

We notice a stable gap between male and female performances at all levels in all marathons, women being on average 10.3%±1.6% (mean ± standard deviation) slower than men (Table S1); mean female winners are 9.9%±1.5% slower than male winners, mean female median is 9.9%±1.6% than male median, and mean female Q1 are 11.1%±1.5% slower that male Q1.

### Correlations

Spearman correlations results are displayed in Table 2, detailed correlations by marathon are available in supplementary data (Table S2).

**Table 2:** Spearman correlations results between all marathons performance levels and environmental parameters: $  =  p<0.1; *  =  p<0.05; **  =  p<0.01; ***  =  p<0.001.

Parameter	Gender	P1	Median	Q1	IQR
Temperature	Women	0.31*	0.30*	0.35**	0.15
	Men	0.48***	0.40***	0.44***	0.25$
Dew Point	Women	0.14	0.18	0.21	0.01
	Men	0.25$	0.19	0.20	0.10
Humidity	Women	−0.3*	−0.16	−0.19	−0.21
	Men	−0.34**	−0.28*	−0.32*	−0.19
Atm. Pressure	Women	0.22$	0.06	0.07	0.06
	Men	0.13	0.04	0.06	0.06
NO2	Women	0.11	0.40**	0.43***	0.33*
	Men	0.25$	0.38**	0.35**	0.27*
O3	Women	0.01	−0.15	−0.11	−0.20
	Men	−0.05	−0.21	−0.24$	−0.11
PM10	Women	0.08	0.15	0.25	0.03
	Men	0.10	0.10	0.09	0.16
SO2	Women	0.21	0.13	0.21	0.02
	Men	0.37**	0.20	0.25$	0.04

P1: first percentile, Q1: first quartile, IQR: Inter Quartile Range.

The environmental parameter that had the most significant correlations with marathons performances was air temperature: it was significantly correlated with all performance levels in both male and female runners.

Humidity was the second parameter with a high impact on performance; it was significantly correlated with women's P1 and men's all performance levels.

The dew point and atmospheric pressure only had a slight influence (p<0.1) in men's P1 and women's P1 respectively, and did not affect the other performance levels.

Concerning the atmospheric pollutants, NO_2_ had the most significant correlation with performance: it was significantly correlated with Q1, IQR and the median for both genders. Sulfur dioxide (SO_2_) was correlated with men's P1 (p<0.01) and had a slight influence (p<0.1) on men's Q1. Finally ozone (O_3_) only had a slight influence (p<0.1) on men's Q1. In the marathon by marathon analysis, ozone (O_3_) had the most significant correlation with performance (Table S2): it was significantly correlated with all performance levels (P1, Q1, IQR and the median) of the Berlin and Boston (except men's IQR) marathon for both genders. It also affected Chicago (men's P1, Q1, and men's median), and New York (women's Q1) marathons.

### Temperature and running speed

**Figure 3:**
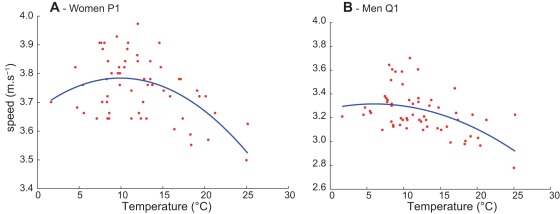
Quadratic second degree polynomial fit for Women's P1 running speeds vs. air temperature, r^2^ = 0.27; p<0.001; max = 9.9°C. B) Men's Q1 running speeds vs. air temperature, r^2^ = 0.24; p<0.001; max = 6°C.

When temperature increased above an optimum, performance decreased. Figure 3 describes the relationship between marathons running speeds and air temperature, fit through a quadratic second degree polynomial curve for women's P1 and men's Q1 of all 60 races.

For each performance level the speed decrease associated with temperature increase and decrease is presented in supplementary data (Table S3).

For example the optimal temperature at which women's P1 maximal running speed was attained was 9.9°C, and an increase of 1°C from this optimal temperature will result in a speed loss of 0.03%. The optimal temperatures to run at maximal speed for men and women, varied from 3.8°C to 9.9°C according to each level of performance (Table S3).

**Figure 4:**
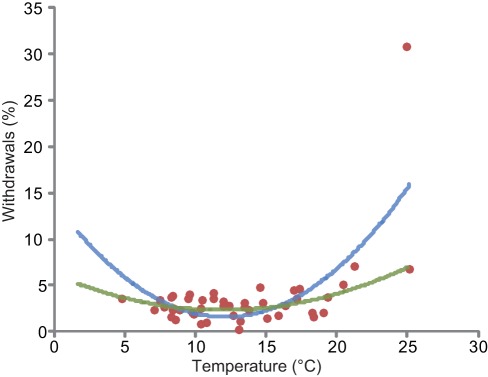
Relationship between air temperature and the percentage of runners' withdrawals, modeled with a quadratic fit (blue curve, r^2^ = 0.36; p<0.0001). The green curve represents the quadratic fit without the maxima (Chicago 2007: 30.74% withdrawals at a race temperature of 25°C).

Warmer air temperatures were associated with higher percentages of runners' withdrawal during a race (Figure 4). After testing linear, quadratic, exponential and logarithmic fits, the quadratic equation was the best fit (r^2^ = 0.36; p<0.0001) for modelling the percentage of runners withdrawals associated with air temperature (Figure 4):
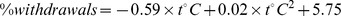



## Discussion

Our study is the first to our knowledge to analyse the exhaustiveness of all marathon finishers' performances in the three major European (Berlin, Paris and London, which were not previously analysed) and three American marathons. Previous studies have mostly analysed American marathons including Chicago, Boston and New York that are analysed in the present paper [9], [11]–[15], but they have only included the performances of the top 3 males and females finishers as well as the 25^th^-, 100^th^-, and 300^th^- place finishers [11], [13]–[15]. In the present study we analysed the total number of finishers in order to exhaustively quantify the effect of climate on runners from all performance levels. Updating and extending earlier results, this study still concludes that the main environmental factor influencing marathon performance remains temperature. The pattern of performance reduction with increasing temperature is analogous in men and women, suggesting no apparent gender differences. In addition the mean gap between male and female performances is the same across all marathons and all performance levels (Table 1). This is consistent with our previous work that showed that the gender gap in athletic performance has been stable for more than 25 years, whatever the environmental conditions [25].

The more the temperature increases, the larger the decreases in running speeds (Table S3). This is supported by the increased percentage of runners' withdrawals when races were contested in very hot weather (Figure 4), and by the significant shift of the race's results through the whole range of performance distribution (Figure 2). The significant effect of air temperature on the median values (Table 2) also suggests that all runners' performances are similarly affected by an increase in air temperature, as seen in Figure 2 showing performances distribution of races in Paris and Chicago with different air temperatures: the significant shift of performance towards the right concerns all runners categories, from the elite to the less trained competitors. In addition the percentage of runner's withdrawals in Chicago 2007 was the highest (30.74%) among all 60 studied races (Figure 1 and Figure 4). Roberts [26] reported that organisers tried to interrupt the race 3.5 h after the start. This was not successful as most of the finishers crossed the finish line much later (up to 7 h after the start); 66 runners were admitted to the hospital (12 intensive care cases with hydration disorders, heat shock syndromes and 1 death). During the 2004 Boston Marathon (T° = 22.5°C) more than 300 emergency medical calls were observed, consequently the race's start time changed from noon to 10 am in order to decrease heat stress and related casualties [26]. The 2007 London Marathon was hot by London standards (air T°  = 19.1°C vs. an average of 11.6°C for the nine other years analysed in our study), 73 hospitalisations were recorded with 6 cases of severe electrolyte imbalance and one death, the total average time (all participants' average) was 17 min slower than usual. In contrast, the number of people treated in London 2008 in cool and rainy conditions (T° = 9.9°C), was 20% lower [26]. Our results showed that the percentage of runners' withdrawals from races significantly increases with increasing temperature (Figure 4). The acceptable upper limit for competition judged by the American College of Sports Medicine (ACSM) is a WBGT of 28°C, but it may not reflect the safety profile of unacclimatized, non-elite marathon runners [3], [26]–[28]. Roberts [26] stated that marathons should not be allowed to start for non-elite racers at a WBGT of 20.5°C. Our results suggest that there is no threshold but a continuous process on both side of an optimum: the larger the gap from the optimal temperature, the lower the tolerance and the higher the risk. In fact, in environments with high heat and humidity, not only is performance potentially compromised, but health is also at risk [29]; both are similarly affected. As soon as WBGT is higher than 13°C the rate of finish line medical encounters and on-course marathon dropouts begin to rise [26] as similarly seen in our study in Figure 4.

Warm weather enhances the risk of exercise induced hyperthermia; its first measurable impact is the reduction of physical performance [4], [14], [29]–[31] as it is detrimental for the cardiovascular, muscular and central nervous systems [32], [33]. More recent work suggested that central fatigue develops before any elevation in body temperature occurs: evidence supported that subjects would subconsciously reduce their velocity earlier after the start of an exercise in hot environment, when internal temperatures are still lower than levels associated with bodily harm. Exercise is thus homeostatically regulated by the decrease of exercise intensity (decrease of running performance and heat production) in order to prevent hyperthermia and related catastrophic failures [34], [35]. On the other hand, cool weather is associated with an improved ability to maintain running velocity and power output as compared to warmer conditions, but very cold conditions also tend to reduce performance [29], [36], [37].

Among the studied races' winners, men's marathon world record was beaten in Berlin in 2007 and 2008 (Haile Gebrselassie in 02:03:59), as well as women's marathon world record, beaten in London 2003 (Paula Radcliffe in 02:15:25). The winners' speeds couldn't be affected in the same way than the other runners by air temperature and the other environmental parameters, because top performances can fluctuate from year to year due to numerous factors, such as prize money, race strategies, or overall competition [11]. Another explanation is that, in all of our 60 studied races, 89.5% of male winners were of African origin (57.9% from Kenya; 21.1% from Ethiopia; and 10.5% from Eritrea, Morocco and South Africa); as well as 54.5% of female winners (27.3% from Kenya and 27.3% from Ethiopia- data not shown). African runners might have an advantage over Caucasian athletes, possibly due to a unique combination of the main endurance factors such as maximal oxygen uptake, fractional utilization of VO_2max_ and running economy [38]. They might also perform better in warm environments as they are usually thinner than Caucasian runners (smaller size and body mass index) producing less heat with lower rates of heat storage [38]–[40]. Psychological factors may also play a role; some hypothesis suggested that regardless of the possible existence of physiological advantages in East African runners, belief that such differences exist may create a background that can have significant positive consequences on performance [41], [42].

Genetics and training influence the tolerance for hyperthermia [4], [38], [43]. Acclimatisation involving repeated exposures to exercise in the heat also results in large improvements in the time to fatigue. Optimal thermoregulatory responses are observed in runners who have been acclimatized to heat and who avoid thirst before and during the race. Their best performances might be less influenced by temperature as winners had been more acclimatized to it [4], [29], [30], [44]. The avoidance of thirst sensation rather than optimum hydration prevents the decline in running performance [45]; contradicting the idea that dehydration associated with a body weight loss of 2% during an exercise will impair performance, recent studies reported that Haile Gebrselassie lost 10% of his body weight when he established his world record [45]–[47].

Previous studies suggested that the impact of weather on speed might depend on running ability, with faster runners being less limited than slower ones [6], [13], [14], [29]. This could be attributable to a longer time of exposition to the environmental conditions of slower runners during the race [11]. Also, slower runners tend to run in closer proximity to other runners with clustering formation [48], [49], which may cause more heat stress as compared with running solo [50]. These elements, however, are not supported after analyzing the full range of finisher's data; at a population level, temperature causes its full effect whatever the initial capacity. Differences in fitness relative to physiological potential may also contribute to differences in performance times and ability to cope with increasing heat stress [11], [48], [49].

There was a strong correlation of running speed with air temperature (Figure 3). The maximal average speeds were performed at an optimal temperature comprised between 3.8°C and 9.9°C depending on the performance level (Table S3); small increases in air temperatures caused marathon performances to decline in a predictable and quantifiable manner. On the other hand, large decreases in air temperatures under the optimum also reduce performances. These optimal temperatures found in the present study are comprised in the optimal temperature range of 5–10°C WBGT found in previous studies [14]; other studies stated that a weather of 10–12°C WBGT is the norm for fast field performance and reported a decrease of performance with increasing WBGT [12], [27], [51], [52]. Best marathon times and most marathon world records were achieved in cool environmental temperatures (10–15°C) and have been run in the early morning during spring and fall [12]. Analysing Gebrselassie's performances in Berlin reveals that they follow the same trend, with both World Records obtained at the lowest temperatures (14°C in 2007 and 13°C in 2008, *vs*. 18°C in 2009 and 22°C in 2006 when he also won these two races without beating the world record).

The relationship between running speed and air temperature defined in our study (Figure 3) is similar to the relationship found between mortality and air temperature (asymmetrical U-like pattern) in France defined by Laaidi et al [53], where mortality rates increase with the lowest and the highest temperatures. A “thermal optimum” occurs in between, where mortality rates are minimal [53]. The great influence that temperature has on performance is comparable to the influence it has on mortality, suggesting that both sports performance and mortality are thermodynamically regulated. This also emphasizes the utility of prevention programs, the assessment of public health impacts and acclimatization before participating in hot marathons [53]. Similar correlations were also found between temperature and swimming performance in juvenile southern catfish [22], and between increases in summer water temperature and elevated mortality rates of adult sockeye salmon [23]; suggesting that physiological adaptations to temperature, similarly occur in various taxons, but vary within specific limits that depend on species and will modify performances.

### Air pollution and performance

The measured levels of pollution had no impact on performance, except for ozone (Table S2) and NO_2_ (Table 2). Assessing the effect of any single air pollutant separately is not simple; it is not isolated in the inhaled air, but rather combined with other parameters. Therefore any possible influence might probably be due to a combination of components. In addition most marathons are held on Sunday mornings, when urban transport activity and its associated emissions are low, and photochemical reactions driven by solar radiation have not yet produced secondary pollutants such as ozone [9]. This is the most probable explanation to our results, confirming previous studies. Among the air pollutants analysed in the present study, ozone and NO_2_ had the greatest effect on decreasing marathon performances (Table S2). Ozone concentrations on the ground increase linearly with air temperature [7], [8], [10]; thus the effect of ozone in our study may be mainly associated with the temperature effect, as seen in Berlin and Chicago. However ozone and other pollutants effects are known to be detrimental to exercise performance only when exposure is sufficiently high. Many studies showed no effect of air pollutants on sports performance [9]. Some of them showed that PM_2.5_ and aerosol acidity were associated with acute decrements in pulmonary function, but these changes in pulmonary function were unlikely to result in clinical symptoms [54]. Others showed that chronic exposure to mixed pollutants during exercise may result in decreased lung function, or vascular dysfunction, and may compromise performance [55]. During the marathons studied here, concentrations of air pollutants never exceeded the limits set forth by national environmental agencies (US Environmental Protection Agency- EPA; AirParif; European Environmental Agency- EEA) or the levels known to alter lung function in laboratory situations [9].

### Conclusions

Air temperature is the most important factor influencing marathon running performance for runners of all levels. It greatly influences the entire distribution of runners' performances as well as the percentage of withdrawals. Running speed at all levels is linked to temperature through a quadratic model. Any increase or decrease from the optimal temperature range will result in running speed decrease. Ozone also has an influence on performance but its effect might be linked to the temperature impact. The model developed in this study could be used for further predictions, in order to evaluate expected performance variations with changing weather conditions.

## Supporting Information

Table S1 **Time values of different descriptive statistics and their variability by marathon and gender.** ^1^ Value of the described statistic for all performances of all year together, hour:min:sec ^2^ Standard deviation of the described statistic for all performances of each year, hour:min:sec ^3^ IQR: Inter Quartile Range.
(DOCX)

Table S2 Spearman correlations results between each marathon performance levels and environmental parameters: $  =  p<0.1; *  =  p<0.05; **  =  p<0.01; ***  =  p<0.001. P1: first percentile, Q1: first quartile, IQR: Inter Quartile Range.
(DOCX)

Table S3 **Optimal temperatures for maximal running speeds of each level of performance, with speed losses associated with each temperature increase.**
(DOCX)

## References

[1] Lippi G, Favaloro EJ, Guidi GC (2008). The genetic basis of human athletic performance. Why are psychological components so often overlooked?. J Physiol 586(Pt 12):3017; author reply.

[2] Macarthur DG, North KN (2005). Genes and human elite athletic performance.. Hum Genet.

[3] Cheuvront SN, Haymes EM (2001). Thermoregulation and marathon running, biological and environmental influences.. Sports Med.

[4] Kenefick RW, Cheuvront SN, Sawka MN (2007). Thermoregulatory function during the marathon.. Sports Med.

[5] Weather Underground website. Internet weather service. Weather data from each marathon race.. http://www.wunderground.com/history/.

[6] Vihma T (2010). Effects of weather on the performance of marathon runners.. Int J Biometeorol.

[7] Shephard RJ (1984). Athletic performance and urban air pollution.. Can Med Assoc J.

[8] Chimenti L, Morici G, Paterno A, Bonanno A, Vultaggio M (2009). Environmental conditions, air pollutants, and airway cells in runners: A longitudinal field study.. J Sports Sci.

[9] Marr LC, Ely MR (2010). Effect of air pollution on marathon running performance.. Med Sci Sports Exerc.

[10] Lippi G, Guidi GC, Maffulli N (2008). Air pollution and sports performance in Beijing.. Int J Sports Med.

[11] Ely MR, Cheuvront SN, Roberts WO, Montain SJ (2007). Impact of weather on marathon-running performance.. Med Sci Sports Exerc.

[12] Ely MR, Cheuvront SN, Montain SJ (2007). Neither cloud cover nor low solar loads are associated with fast marathon performance.. Med Sci Sports Exerc.

[13] Ely MR, Martin DE, Cheuvront SN, Montain SJ (2008). Effect of ambient temperature on marathon pacing is dependent on runner ability.. Med Sci Sports Exerc.

[14] Montain SJ, Ely MR, Cheuvront SN (2007). Marathon performance in thermally stressing conditions.. Sports Med.

[15] Martin DE, Buoncristiani JF (1999). The effects of temperature on marathon runners' performance.. Chance.

[16] Trapasso LM, Cooper JD (1989). Record performances at the Boston Marathon: biometeorological factors.. Int J Biometeorol.

[17] Online worldwide athletic results database website. Marathons races results.. http://www.athlinks.com.

[18] AirParif website. Air pollution data for Paris retrieved (March – May 2009). Available: http://www.airparif.com

[19] Station Database of the Environmental Agency. Air pollution data for Berlin retrieved (June 24, 2009). Available: http://www.env-it.de/stationen/public/language.dojsessionid=FB278996EE26B0351076A5D974C8BD04?language=en

[20] LondonAir website. Air pollution data for London retrieved (May 26, 2009). Available: http://www.londonair.org.uk/london/asp/default.asp

[21] Berthelot G, Tafflet M, El Helou N, Len S, Escolano S (2010). Athlete atypicity on the edge of human achievement: Performances stagnate after the last peak, in 1988.. PLoS ONE, 5 (1),.

[22] Zeng LQ, Cao ZD, Fu SJ, Peng JL, Wang YX (2009). Effect of temperature on swimming performance in juvenile southern catfish (Silurus meridionalis).. Comp Biochem Physiol A Mol Integr Physiol.

[23] Eliason EJ, Clark TD, Hague MJ, Hanson LM, Gallagher ZS (2011). Differences in thermal tolerance among sockeye salmon populations.. Science.

[24] Kirschbaum MUF, Watt MS (2011). Use of a process-based model to describe spatial variation in Pinus radiate productivity in New Zealand.. Forest Ecology and Management.

[25] Thibault V, Guillaume M, Berthelot G, El Helou N, Schaal K (2010). Women and men in sport performance: the gender gap has not evolved since 1983.. J Sports Sci Med.

[26] Roberts WO (2010). Determining a “do not start” temperature for a marathon on the basis of adverse outcomes.. Med Sci Sports Exerc.

[27] Zhang S, Meng G, Wang Y, Li J (1992). Study of the relationships between weather conditions and the marathon race, and of meteorotropic effects on distance runners.. Int J Biometeorol.

[28] Armstrong LE, Epstein Y, Greenleaf JE, Haymes EM, Hubbard RW (1996). American College of Sports Medicine position stand. Heat and cold illnesses during distance running.. Med Sci Sports Exerc 28(12): i–.

[29] Maughan RJ, Watson P, Shirreffs SM (2007). Heat and cold, what does the environment do to the marathon runner?. Sports Med.

[30] Hargreaves M (2008). Physiological limits to exercise performance in the heat.. J Sci Med Sport.

[31] Walters TJ, Ryan KL, Tate LM, Mason PA (2000). Exercise in the heat is limited by a critical internal temperature.. J Appl Physiol.

[32] Coyle EF (2007). Physiological regulation of marathon performance.. Sports Med.

[33] González-Alonso J (2007). Hyperthermia Impairs Brain, Heart and Muscle Function in Exercising Humans.. Sports Med.

[34] Tucker R, Rauch L, Harley YX, Noakes TD (2004). Impaired exercise performance in the heat is associated with an anticipatory reduction in skeletal muscle recruitment.. Pflugers Arch.

[35] Tucker R, Marle T, Lambert EV, Noakes TD (2006). The rate of heat storage mediates an anticipatory reduction in exercise intensity during cycling at a fixed rating of perceived exertion.. J Physiol 574(Pt.

[36] Nimmo M (2004). Exercise in the cold.. J Sports Sci.

[37] Weller AS, Millard CE, Stroud MA, Greenhaff PL, Macdonald IA (1997). Physiological responses to a cold, wet, and windy environment during prolonged intermittent walking.. Am J Physiol 272(1 Pt.

[38] Larsen HB (2003). Kenyan dominance in distance running.. Comp Biochem Physiol A Mol Integr Physiol.

[39] Marino FE, Lambert MI, Noakes TD (2004). Superior performance of African runners in warm humid but not in cool environmental conditions.. J Appl Physiol.

[40] Marino FE, Mbambo Z, Kortekaas E, Wilson G, Lambert MI (2000). Advantages of smaller body mass during distance running in warm, humid environments.. Pflügers Arch.

[41] Hamilton B (2000). East African running dominance: what is behind it?. Br J Sports Med.

[42] Baker J, Horton S (2003). East African running dominance revisited: a role for stereotype threat?. Br J Sports Med.

[43] Sawka MN Young A 2006 Physiological systems and their responses to conditions of heat and cold. Tipton CM American College of Sports Medicine's Advanced exercise physiology Philadelphia (PA) Lippincott Williams and Wilkins 535–563

[44] Zouhal H, Groussard C, Vincent S, Jacob C, Abderrahman AB (2009). Athletic performance and weight changes during the “Marathon of Sands” in athletes well-trained in endurance.. Int J Sports Med.

[45] Goulet ED (2011). Effect of exercise-induced dehydration on time-trial exercise performance: a meta-analysis.. Br J Sports Med.

[46] Zouhal H, Groussard C, Minter G, Vincent S, Cretual A (2011). Inverse relationship between percentage body weight change and finishing time in 643 forty-two-kilometre marathon runners.. Br J Sports Med.

[47] Beis LY, Wright-Whyte M, Fudge B, Noakes T, Pitsiladis YP (2012). Drinking Behaviors of Elite Male Runners During Marathon Competition.. http://dx.doi.org/10.1097/JSM.0b013e31824a55d7.

[48] Alvarez-Ramirez J, Rodriguez E (2006). Scaling properties of marathon races.. Physica A: Stat Mech Appl.

[49] Alvarez-Ramirez J, Rodriguez E, Dagduga L (2007). Time-correlations in marathon arrival sequences.. Physica A: Stat Mech Appl.

[50] Dawson NJ, De Freitas CR, Mackey WJ, Young AA (1987). The stressful microclimate created by massed fun runners.. Transactions of the Menzies Foundation.

[51] Galloway SDR, Maughan RJ (1997). Effects of ambient temperature on the capacity to perform prolonged cycle exercise in man.. Med Sci Sports Exerc.

[52] Buoncristiani JF, Martin DE (1983). Factors affecting runners' marathon performance.. Chance.

[53] Laaidi M, Laaidi K, Besancenot JP (2006). Temperature-related mortality in France, a comparison between regions with different climates from the perspective of global warming.. Int J Biometeorol.

[54] Korrick SA, Neas LM, Dockery DW, Gold DR, Allen GA (1998). Effects of ozone and other pollutants on the pulmonary function of adult hickers.. Environ Health Perspect.

[55] Rundell KW (2012). Effect of air pollution on athlete health and performance..

