# Supplementary material for: Impact of Environmental Parameters on Marathon Running Performance
Source: PLoS One. 2012 May 23;7(5):e37407. doi: 10.1371/journal.pone.0037407 (PMC3359364; doi:10.1371/journal.pone.0037407)
Supplement: Table S1 — Time values of different descriptive statistics and their variability by marathon and gender. 1 Value of the described statistic for all performances of all year together, hour:min:sec 2 Standard deviation of the described statistic for all performances of each year, hour:min:sec 3 IQR: Inter Quartile Range. (DOCX) [file pone.0037407.s001.docx]

|  |  | **Women** | | **Men** | |
| --- | --- | --- | --- | --- | --- |
| **Marathon** | **Statistic**  **(performance level)** | **Value^1^** | **Std dev^2^** | **Value^1^** | **Std dev^2^** |
|  |  | **2001-2010** | **of 2001-2010** | **2001-2010** | **of 2001-2010** |
| **Berlin** | Number of finishers | 59233 | 1182 | 247788 | 2516 |
|  | Winner | 02:19:12 | 00:02:34 | 02:03:59 | 00:01:30 |
|  | Last finisher | 07:09:24 | 00:22:40 | 07:42:28 | 00:25:09 |
|  | Median | 04:26:10 | 00:04:16 | 03:58:12 | 00:04:28 |
|  | IQR^3^ | 00:51:02 | 00:01:53 | 00:52:44 | 00:02:29 |
| **Boston** | Number of finishers | 72364 | 1647 | 113691 | 1677 |
|  | Winner | 02:20:43 | 00:03:10 | 02:05:52 | 00:02:23 |
|  | Last finisher | 07:41:09 | 00:40:02 | 07:49:11 | 00:47:11 |
|  | Median | 03:57:18 | 00:07:33 | 03:38:41 | 00:09:29 |
|  | IQR | 00:40:55 | 00:03:14 | 00:48:47 | 00:03:39 |
| **Chicago** | Number of finishers | 135443 | 1708 | 182493 | 1271 |
|  | Winner | 02:17:18 | 00:04:49 | 02:05:41 | 00:01:43 |
|  | Last finisher | 08:47:25 | 00:27:16 | 09:26:05 | 00:42:53 |
|  | Median | 04:42:05 | 00:11:20 | 04:15:06 | 00:15:09 |
|  | IQR | 01:05:23 | 00:05:18 | 01:07:59 | 00:05:39 |
| **Londres** | Number of finishers | 96686 | 1728 | 239120 | 694 |
|  | Winner | 02:15:25 | 00:02:55 | 02:05:10 | 00:01:04 |
|  | Last finisher | 10:17:12 | 01:00:34 | 10:20:47 | 00:58:03 |
|  | Median | 04:45:49 | 00:05:28 | 04:16:08 | 00:05:28 |
|  | IQR | 01:10:23 | 00:03:29 | 01:07:11 | 00:03:18 |
| **New York** | Number of finishers | 120879 | 2529 | 243051 | 3532 |
|  | Winner | 02:22:31 | 00:02:09 | 02:07:43 | 00:00:52 |
|  | Last finisher | 10:22:47 | 00:43:37 | 09:59:58 | 00:31:56 |
|  | Median | 04:40:28 | 00:04:54 | 04:16:39 | 00:06:38 |
|  | IQR | 01:02:40 | 00:01:38 | 01:02:08 | 00:01:22 |
| **Paris** | Number of finishers | 43030 | 836 | 237293 | 2223 |
|  | Winner | 02:22:02 | 00:02:00 | 02:05:47 | 00:01:13 |
|  | Last finisher | 06:52:51 | 00:11:56 | 06:52:46 | 00:11:27 |
|  | Median | 04:32:37 | 00:06:19 | 03:58:58 | 00:05:25 |
|  | IQR | 00:53:02 | 00:02:21 | 00:55:39 | 00:03:18 |

**Supplementary Table1**–Time values of different descriptive statistics and their variability by marathon and gender

^1^ Value of the described statistic for all performances of all year together, hour:min:sec

^2^ Standard deviation of the described statistic for all performances of each year, hour :min:sec

^3^ IQR: Inter Quartile Range
