# Supplementary material for: Impact of Environmental Parameters on Marathon Running Performance
Source: PLoS One. 2012 May 23;7(5):e37407. doi: 10.1371/journal.pone.0037407 (PMC3359364; doi:10.1371/journal.pone.0037407)
Supplement: Table S2 — Spearman correlations results between each marathon performance levels and environmental parameters: $ = p<0.1; * = p<0.05; ** = p<0.01; *** = p<0.001. P1: first percentile, Q1: first quartile, IQR: Inter Quartile Range. (DOCX) [file pone.0037407.s002.docx]

| **Marathon** | **Parameter** | **Gender** | **P1** | **Median** | **Q1** | **IQR** | **Marathon** | **Parameter** | **Gender** | **P1** | **Median** | **Q1** | **IQR** |
| --- | --- | --- | --- | --- | --- | --- | --- | --- | --- | --- | --- | --- | --- |
| **Berlin** | Temperature | Female | 0,56$ | 0,60$ | 0,71* | 0,68* | **Boston** | Temperature | Female | 0.38 | 0.44 | 0.46 | 0.45 |
|  |  | Male | 0.60$ | 0.78** | 0.82** | 0.74* |  |  | Male | 0.25 | 0.49 | 0.46 | 0.46 |
|  | Dew Point | Female | 0.35 | 0.44 | 0.41 | 0,64* |  | Dew Point | Female | 0.03 | -0.01 | -0.06 | 0.08 |
|  |  | Male | 0.15 | 0.36 | 0.35 | 0.47 |  |  | Male | -0.01 | -0.05 | -0.06 | 0.06 |
|  | Humidity | Female | -0.34 | -0,70* | -0,74* | -0.51 |  | Humidity | Female | -0.52 | -0,58$ | -0,63$ | -0.50 |
|  |  | Male | -0.47 | -0.83** | -0.82** | -0.75* |  |  | Male | -0.57$ | -0.62$ | -0.63$ | -0.52 |
|  | Atm. Pressure | Female | -0,57$ | -0.23 | -0.28 | 0.08 |  | Atm. Pressure | Female | 0.07 | 0.12 | 0.12 | 0.10 |
|  |  | Male | -0.26 | -0.03 | -0.07 | 0.26 |  |  | Male | 0.01 | 0.13 | 0.12 | 0.12 |
|  | NO2 | Female | 0.24 | 0,63$ | 0.51 | 0.53 |  | NO2 | Female | 0.09 | 0.01 | 0.02 | -0.01 |
|  |  | Male | 0.08 | 0.65* | 0.55$ | 0.82** |  |  | Male | 0.15 | 0.02 | 0.02 | 0.33 |
|  | O3 | Female | 0,55$ | 0,74* | 0,75* | 0,68* |  | O3 | Female | 0,71* | 0,76* | 0,76* | 0,68* |
|  |  | Male | 0.61$ | 0.87** | 0.84** | 0.84** |  |  | Male | 0.75* | 0.75* | 0.76* | 0.50 |
|  | PM10 | Female | 0.28 | 0.07 | 0.17 | 0.17 |  | PM10 | Female | . | . | . | . |
|  |  | Male | 0.16 | 0.14 | 0.19 | 0.41 |  |  | Male | . | . | . | . |
|  | SO2 | Female | 0.30 | 0.18 | 0.23 | 0.12 |  | SO2 | Female | 0.03 | 0.02 | 0.05 | 0.08 |
|  |  | Male | 0.02 | 0.27 | 0.27 | 0.46 |  |  | Male | 0.10 | 0.08 | 0.05 | 0.46 |
| **Chicago** | Temperature | Female | 0.64* | 0.73* | 0.89*** | 0.36 | **London** | Temperature | Female | 0.75* | 0.55$ | 0.71* | 0.36 |
|  |  | Male | 0.80** | 0.79** | 0.84** | 0.48 |  |  | Male | 0.39 | 0.64* | 0.68* | 0.56$ |
|  | Dew Point | Female | 0.48 | 0.76* | 0.92*** | 0.44 |  | Dew Point | Female | -0.01 | 0.27 | 0.29 | 0.05 |
|  |  | Male | 0.66* | 0.79** | 0.85** | 0.61$ |  |  | Male | -0.43 | 0.05 | 0.13 | 0.10 |
|  | Humidity | Female | 0.03 | 0.23 | 0.33 | 0.01 |  | Humidity | Female | -0.56$ | -0.28 | -0.36 | -0.34 |
|  |  | Male | -0.01 | 0.23 | 0.29 | 0.16 |  |  | Male | -0.70* | -0.49 | -0.45 | -0.44 |
|  | Atm. Pressure | Female | 0.30 | -0.22 | -0.39 | -0.21 |  | Atm. Pressure | Female | 0.47 | 0.45 | 0.57$ | 0.34 |
|  |  | Male | 0.10 | -0.23 | -0.31 | -0.39 |  |  | Male | 0.20 | 0.44 | 0.41 | 0.43 |
|  | NO2 | Female | -0.07 | 0.01 | -0.07 | 0.13 |  | NO2 | Female | -0.13 | -0.02 | -0.12 | -0.08 |
|  |  | Male | -0.10 | -0.13 | -0.15 | 0.19 |  |  | Male | -0.45 | -0.31 | -0.28 | 0.05 |
|  | O3 | Female | 0.46 | 0.50 | 0.66* | 0.43 |  | O3 | Female | -0.20 | 0.09 | 0.10 | 0.28 |
|  |  | Male | 0.70* | 0.66* | 0.72* | 0.43 |  |  | Male | 0.47 | 0.26 | 0.22 | 0.07 |
|  | PM10 | Female | . | . | . | . |  | PM10 | Female | . | . | . | . |
|  |  | Male | . | . | . | . |  |  | Male | . | . | . | . |
|  | SO2 | Female | 0.90*** | 0.47 | 0.64$ | 0.11 |  | SO2 | Female | 0.06 | 0.15 | 0.21 | 0.30 |
|  |  | Male | 0.88** | 0.58 | 0.65$ | 0.18 |  |  | Male | 0.36 | 0.07 | 0.06 | 0.42 |
| **New York** | Temperature | Female | 0.49 | 0.64* | 0.62$ | -0.35 | **Paris** | Temperature | Female | 0.54 | 0.45 | 0.51 | 0.25 |
|  |  | Male | 0.37 | 0.78** | 0.68* | 0.53 |  |  | Male | 0.57$ | 0.51 | 0.54 | 0.40 |
|  | Dew Point | Female | 0.57$ | 0.56$ | 0.65* | -0.57$ |  | Dew Point | Female | 0.33 | 0.39 | 0.34 | 0.41 |
|  |  | Male | 0.45 | 0.78** | 0.71* | 0.43 |  |  | Male | 0.36 | 0.42 | 0.39 | 0.40 |
|  | Humidity | Female | 0.70* | 0.53 | 0.64* | -0.59$ |  | Humidity | Female | 0.20 | 0.26 | 0.10 | 0.47 |
|  |  | Male | 0.56$ | 0.69* | 0.68* | 0.39 |  |  | Male | 0.17 | 0.40 | 0.33 | 0.35 |
|  | Atm. Pressure | Female | -0.09 | -0.40 | -0.40 | 0.21 |  | Atm. Pressure | Female | 0.02 | 0.02 | -0.10 | -0.05 |
|  |  | Male | -0.20 | -0.62$ | -0.60$ | -0.16 |  |  | Male | -0.10 | -0.11 | -0.05 | -0.20 |
|  | NO2 | Female | 0.53 | 0.74* | 0.79* | 0.12 |  | NO2 | Female | -0.04 | 0.14 | 0.26 | 0.25 |
|  |  | Male | 0.54 | 0.68* | 0.71* | 0.16 |  |  | Male | 0.01 | 0.27 | 0.11 | 0.28 |
|  | O3 | Female | -0.22 | -0.49 | -0.59$ | 0.28 |  | O3 | Female | -0.14 | 0.12 | 0.25 | 0.06 |
|  |  | Male | -0.26 | -0.37 | -0.41 | 0.14 |  |  | Male | -0.04 | -0.06 | -0.13 | 0.09 |
|  | PM10 | Female | . | . | . | . |  | PM10 | Female | 0.46 | 0.23 | 0.38 | 0.12 |
|  |  | Male | . | . | . | . |  |  | Male | 0.47 | 0.49 | 0.52 | 0.29 |
|  | SO2 | Female | 0.15 | 0.52 | 0.52 | 0.29 |  | SO2 | Female | -0.66* | -0.53 | -0.50 | -0.30 |
|  |  | Male | 0.38 | 0.45 | 0.55 | -0.11 |  |  | Male | -0.67* | -0.56$ | -0.63* | -0.45 |

**Supplementary Table2**–Spearman correlations results between each marathon performance levels and environmental parameters: $ = p<0.1; * = p<0.05; ** = p<0.01; *** = p<0.001. P1: first percentile, Q1: first quartile, IQR: Inter Quartile Range.
