# Supplementary material for: Impact of Environmental Parameters on Marathon Running Performance
Source: PLoS One. 2012 May 23;7(5):e37407. doi: 10.1371/journal.pone.0037407 (PMC3359364; doi:10.1371/journal.pone.0037407)
Supplement: Table S3 — Optimal temperatures for maximal running speeds of each level of performance, with speed losses associated with each temperature increase. (DOCX) [file pone.0037407.s003.docx]

| **Performance**  **Level** | **Men** | | | | **Women** | | | |
| --- | --- | --- | --- | --- | --- | --- | --- | --- |
|  | **T°** | **°C** | **Speed**  **(m.s^-1^)** | **Speed loss**  **(%)** | **T°** | **°C** | **Speed**  **(m.s^-1^)** | **Speed loss**  **(%)** |
| **P1** | Peak-10° | -6.19 | 4.29 | 1.41 | Peak-10° | -0.09 | 3.67 | 2.97 |
|  | Peak-5° | -1.19 | 4.34 | 0.35 | Peak-5° | 4.91 | 3.76 | 0.74 |
|  | Peak | 3.81 | 4.36 | 0 | peak | 9.91 | 3.78 | 0 |
|  | peak+5° | 8.81 | 4.34 | 0.36 | peak+5° | 14.91 | 3.76 | 0.75 |
|  | peak+10° | 13.81 | 4.29 | 1.44 | peak+10° | 19.91 | 3.67 | 3.06 |
|  | peak+15° | 18.81 | 4.22 | 3.29 | peak+15° | 24.91 | 3.53 | 7.16 |
|  | peak+20° | 23.81 | 4.11 | 6 | peak+20° | 29.91 | 3.34 | 13.47 |
| **Median** | Peak-10° | -3.76 | 2.85 | 3.77 | Peak-10° | -3.25 | 2.58 | 2.76 |
|  | Peak-5° | 1.24 | 2.93 | 0.94 | Peak-5° | 1.75 | 2.63 | 0.69 |
|  | Peak | 6.24 | 2.96 | 0 | peak | 6.75 | 2.65 | 0 |
|  | peak+5° | 11.24 | 2.94 | 0.95 | peak+5° | 11.75 | 2.63 | 0.70 |
|  | peak+10° | 16.24 | 2.85 | 3.91 | peak+10° | 16.75 | 2.58 | 2.84 |
|  | peak+15° | 21.24 | 2.71 | 9.26 | peak+15° | 21.75 | 2.49 | 6.63 |
|  | peak+20° | 26.24 | 2.52 | 17.73 | peak+20° | 26.75 | 2.36 | 12.43 |
| **Q1** | Peak-10° | -3.99 | 3.21 | 3.27 | Peak-10° | -3.15 | 2.85 | 2.51 |
|  | Peak-5° | 1.01 | 3.29 | 0.82 | Peak-5° | 1.85 | 2.91 | 0.63 |
|  | Peak | 6.02 | 3.32 | 0 | peak | 6.85 | 2.93 | 0 |
|  | peak+5° | 11.02 | 3.29 | 0.82 | peak+5° | 11.85 | 2.91 | 0.63 |
|  | peak+10° | 16.02 | 3.21 | 3.38 | peak+10° | 16.85 | 2.85 | 2.58 |
|  | peak+15° | 21.02 | 3.07 | 7.93 | peak+15° | 21.85 | 2.76 | 6.00 |
|  | peak+20° | 26.02 | 2.88 | 15.03 | peak+20° | 26.85 | 2.63 | 11.18 |
| **Q3** | Peak-10° | -2.58 | 2.51 | 4.41 | Peak-10° | -2.65 | 2.32 | 3.04 |
|  | Peak-5° | 2.42 | 2.59 | 1.10 | Peak-5° | 2.35 | 2.37 | 0.76 |
|  | Peak | 7.42 | 2.62 | 0 | peak | 7.35 | 2.39 | 0 |
|  | peak+5° | 12.42 | 2.59 | 1.12 | peak+5° | 12.35 | 2.37 | 0.77 |
|  | peak+10° | 17.42 | 2.51 | 4.61 | peak+10° | 17.35 | 2.32 | 3.14 |
|  | peak+15° | 22.42 | 2.36 | 11.01 | peak+15° | 22.35 | 2.23 | 7.35 |
|  | peak+20° | 27.42 | 2.16 | 21.42 | peak+20° | 27.35 | 2.10 | 13.85 |

**Supplementary Table 3**–Optimal temperatures for maximal running speeds of each level of performance, with speed losses associated with each temperature increase.
